# Physical fitness and physical activity association with cognitive function and quality of life: baseline cross-sectional analysis of the PREDIMED-Plus trial

**DOI:** 10.1038/s41598-020-59458-6

**Published:** 2020-02-26

**Authors:** Lidia Daimiel, Miguel A. Martínez-González, Dolores Corella, Jordi Salas-Salvadó, Helmut Schröder, Jesús Vioque, Dora Romaguera, J. Alfredo Martínez, Julia Wärnberg, Jose Lopez-Miranda, Ramón Estruch, Naomi Cano-Ibáñez, Angel Alonso-Gómez, Josep A. Tur, Francisco J. Tinahones, Lluis Serra-Majem, Rafael M. Micó-Pérez, José Lapetra, Alba Galdón, Xavier Pintó, Josep Vidal, Victor Micó, Gonzalo Colmenarejo, José J. Gaforio, Pilar Matía, Emilio Ros, Pilar Buil-Cosiales, Zenaida Vázquez-Ruiz, José V. Sorlí, Indira Paz Graniel, Aida Cuenca-Royo, Cristina Gisbert-Sellés, Aina M. Galmes-Panades, M. Angeles Zulet, Antonio García-Ríos, Andrés Díaz-López, Rafael de la Torre, Iñigo Galilea-Zabalza, José M. Ordovás

**Affiliations:** 1 Nutritional Genomics and Epigenomics Group, IMDEA Food, CEI UAM + CSIC, Madrid, Spain; 2 CIBER Fisiopatología de la Obesidad y Nutrición (CIBEROBN), Instituto de Salud Carlos III (ISCIII), Madrid, Spain; 3 Department of Preventive Medicine and Public Health, University of Navarra, IDISNA, Pamplona, Spain; 4 Department of Nutrition, Harvard T. H. Chan School of Public Health, Boston, USA; 5 Department of Preventive Medicine, University of Valencia, Valencia, Spain; 6 Universitat Rovira i Virgili, Departament de Bioquímica i Biotecnologia, Unitat de Nutrició. Hospital Universitari San Joan de Reus, Institut d’Investigació Pere Virgili (IISPV), Reus, Spain; 7 CIBER Epidemiología y Salud Pública (CIBERESP), Instituto de Salud Carlos III (ISCIII), Madrid, Spain; 8 Unit of Cardiovascular Risk and Nutrition, Institut Hospital del Mar de Investigaciones Médicas Municipal d’Investigació Mèdica (IMIM), Barcelona, Spain; 9 Instituto de Investigación Sanitaria y Biomédica de Alicante, ISABIAL, Alicante, Spain; 10 Health Research Institute of the Balearic Islands (IdISBa), Palma de Mallorca, Spain; 11 Department of Nutrition, Food Sciences, and Physiology, University of Navarra, Pamplona, Spain; 12 Cardiometabolic Nutrition Group, IMDEA Food, CEI UAM + CSIC, Madrid, Spain; 13 Department of Nursing, School of Health Sciences, University of Málaga, Instituto de Investigación Biomédica de Málaga (IBIMA), Málaga, Spain; 14 Lipids and Atherosclerosis Unit, Department of Internal Medicine, Maimonides Biomedical Research Institute of Cordoba (IMIBIC), Reina Sofia University Hospital, University of Cordoba, Córdoba, Spain; 15 Department of Internal Medicine, IDIBAPS, Hospital Clinic, University of Barcelona, Barcelona, Spain; 16 Department of Preventive Medicine, University of Granada, Granada, Spain; 17 Bioaraba Health Research Institute; Osakidetza Basque Health Service, Araba University Hospital; University of the Basque Country UPV/EHU, Vitoria-Gasteiz, Spain; 18 Research Group on Community Nutrition & Oxidative Stress, University of Balearic Islands, Palma de Mallorca, Spain; 19 Instituto de Investigación Biomédica de Málaga (IBIMA), Virgen de la Victoria Hospital, Department of Endocrinology, University of Málaga, Málaga, Spain; 20 Research Institute of Biomedical and Health Sciences (IUIBS), University of Las Palmas de Gran Canaria, Preventive Medicine Service, Centro Hospitalario Universitario Insular Materno Infantil (CHUIMI), Canarian Health Service., Las Palmas, Spain; 21 Cátedra cronicidad Universidad Miguel Hernández, Fundación, Semergen Spain; 22 Department of Family Medicine, Research Unit, Distrito Sanitario Atención Primaria Sevilla, Sevilla, Spain; 23 Department of Endocrinology, Fundación Jiménez-Díaz, Madrid, Spain; 24 Lipids and Vascular Risk Unit, Internal Medicine, Hospital Universitario de Bellvitge, Hospitalet de Llobregat, Barcelona, Spain; 25 CIBER Diabetes y enfermedades metabólicas (CIBERDEM), Instituto de Salud Carlos III (ISCIII), Madrid, Spain; 26 Department of Endocrinology, IDIBAPS, Hospital Clinic, University of Barcelona, Barcelona, Spain; 27 Biostatistics and Bioinformatics Unit, IMDEA Food, CEI UAM+CSIC, Madrid, Spain; 28 Center for Advanced Studies in Olive Grove and Olive Oils, University of Jaén, Jaén, Spain; 29 Department of Endocrinology and Nutrition, Instituto de Investigación Sanitaria Hospital Clínico San Carlos (IdISSC), Madrid, Spain; 30 Primary Health Care. Servicio Navarro de Salud, Pamplona, Spain; 31 Integrative Pharmacology and Systems Neurosciences Institut Hospital del Mar de Investigaciones Médicas Municipal d’Investigació Mèdica (IMIM), Barcelona, Spain; 32 Primary Health Care Center San Vicente del Raspeig, Alicante, Spain; 33 Nutrition and Genomics Laboratory, JM_USDA Human Nutrition Research Center on Aging, Tufts University, Boston, Massachusetts 02111 USA; 34 Department of Cardiovascular Epidemiology and Population Genetics, Centro Nacional de Investigaciones Cardiovasculares (CNIC), Madrid, Spain

**Keywords:** Epidemiology, Quality of life

## Abstract

Physical activity (PA) has been hypothesized to be effective to maintaining cognitive function and delay cognitive decline in the elderly, but physical fitness (PF) could be a better predictor of cognitive function. We aimed to study the association between PA and PF with cognitive function and quality of life using cross-sectional data from 6874 participants of the PREDIMED-Plus trial (64.9 ± 4.9 years, 48.5% female). PF and PA were measured with a Chair Stand Test, the REGICOR and Rapid Assessment Physical Activity questionnaires. Cognitive function was measured with Mini-mental State Examination, Control Oral Word Association Test, Trail Making Test and Digit Span tests; whereas health-related quality of life was assessed with the SF36-HRQL test. Cognitive and quality of life scores were compared among PF quartiles and PA levels (low, moderate and high) with ANCOVA and with Chair Stand repetitions and energy expenditure from total PA with multivariable linear regression adjusted for confounding factors. PF associated with higher scores in phonemic and semantic verbal fluency tests and with lower TMT A time. However, PA was not associated with the neurocognitive parameters evaluated. Both PF and PA levels were strongly associated with a better quality of life. We concluded that PF, but not PA, is associated with a better cognitive function. This trial was retrospectively registered at the International Standard Randomized Controlled Trial (ISRCTN89898870, https://www.isrctn.com/ISRCTN89898870?q=ISRCTN89898870&filters=&sort=&offset=1&totalResults=1&page=1&pageSize=10&searchType=basic-search) on 07/24/2014.

## Introduction

Aging traditionally involves a decline in executive functions, which in some cases progresses towards cognitive impairment. Given that the world’s population is getting older, the prevalence of subjective cognitive decline and mild cognitive impairment (MCI) is on the rise^1,2^. Therefore, the development of strategies to maintain normal cognitive function and delay cognitive decline is relevant.

Cardiovascular risk factors like type 2 diabetes (T2DM) or obesity, have been consistently associated with cognitive decline and MCI^2,3^, as well as the progression to dementia^4^. Thus, it has been postulated that therapeutic management of these factors can be useful to maintain cognitive function and even slow down cognitive decline^2^. Therapies based on lifestyle interventions, specifically those designed to prevent cardiovascular disease, could be a safe approach to maintain cognitive function and delay aging-associated decline. Although approaches using dietary supplements did not report significant benefits^5,6^, studies with traditional Mediterranean Diet (MedDiet) have shown promising results^7–10^. MedDiet has also been reported to be associated with better self-reported quality of life^11^.

Physical activity (PA) is another modifiable lifestyle factor with potentially large beneficial effects on cognitive functioning. It has been postulated that exercise promotes healthy aging and has beneficial effects on cognition in the elderly^12^. Several cross-sectional analyses and interventional studies have found positive associations between PA, especially aerobic exercise, and cognitive function in the elderly^13–16^. These studies strongly suggest that the PA association with cognitive function is relevant enough to be taken into consideration when designing lifestyle interventions addressed to improve health in the elderly. However, reported associations between PA and cognitive health are often small to moderate, depending on the cognitive domain, and inconsistent across studies^15–23^. PA has also been associated with a better subjective quality of life^24,25^.

Although there is compelling evidence pointing towards an association between PA and cognitive function, the heterogeneity of the findings calls for additional research to unravel those conditions where the effect is beneficial. These inconsistencies can be due to the different baseline health status of participants, environmental factors, tools used to assess cognition and PA, different PA programs, or statistical models. In this regard, physical fitness (PF) needs to be considered. PF is a measure of physical condition and well-being, and it has been reported that PA with an effective improvement in cardiovascular fitness has a greater effect on cognitive function than PA focused on strength^26^. We aimed to investigate the relationship between PA and PF with cognitive function and quality of life, independently of the impact of the adherence to an energy-restricted MedDiet, using baseline data from the PREDIMED-Plus trial^27^.

## Results

### Association between PF and cognitive function

Participants in the higher physical PF quartile (Q4) were mainly younger males (Table 1). Higher PF was associated with lower BMI (p < 0.001), longer sleeping time at weekends (p = 0.027), lengthier schooling time (p < 0.001) and higher education (p < 0.001). Former smokers were also more frequently classified in the Q4 (p < 0.001) as well as married participants (p < 0.001) and active workers (p < 0.001) (Table 1). Moreover, participants in the highest PF quartile had a lower prevalence of diabetes (p < 0.001), depression (p < 0.001) and hypertension (p = 0.002) (Table 1).

**Table 1:** Baseline characteristics of the participants according to baseline fitness and PA levels.

	Total	Chair stand test quartile					Physical Activity Level			
		Q1 (n = 2340)	Q2 (n = 1493)	Q3 (n = 1631)	Q4 (n = 1410)	p value	Light (n = 4118)	Moderate (n = 1297)	High (n = 1459)	p value
**N**	6874	2340	1493	1631	1410		4118	1297	1459	
**Age (years)**	64.95 ± 4.92	65.84 ± 4.78	64.98 ± 4.82	64.66 ± 5.01	63.79 ± 4.86	**<0.001**	65.02 ± 4.99	64.81 ± 4.77	64.88 ± 4.83	NS
**Female sex (%)**	48.52	59.91	48.29	44.08	34.96	**<0.001**	52.19	42.87	43.18	**<0.001**
**Weight (Kg)**	86.59 ± 13	87.06 ± 13.1	87.07 ± 13.29	85.89 ± 12.88	86.1 ± 12.6	**0.008**	87.32 ± 13.28	86.14 ± 12.85	84.9 ± 12.11	**<0.001**
**BMI (Kg/m**^**2**^**)**	32.56 ± 3.45	33.25 ± 3.53	32.63 ± 3.49	32.14 ± 3.32	31.84 ± 3.2	**<0.001**	33.02 ± 3.55	32.11 ± 3.26	31.67 ± 3.08	**<0.001**
**Smoking status (%)**						**<0.001**				
**Current smoker**	12.53	11.97	14	13.67	10.57		14.21	10.72	9.39	**<0.001**
**Former smoker**	43.58	37.78	43	44.27	53.05		39.92	48.57	49.49	
**Never smoker**	43.89	50.26	43	42.06	36.38		45.87	40.71	41.12	
**Adherence to er-MedDiet**	8.5 ± 2.67	8.44 ± 2.59	8.54 ± 2.69	8.53 ± 2.69	8.51 ± 2.75	NS	8.25 ± 2.59	8.64 ± 2.67	9.06 ± 2.8	**<0.001**
**Marital status (%)**						**<0.001**				**0.018**
**Married**	76.51	74.15	76.16	77.87	79.22		75.33	77.1	79.3	
**Single**	5.14	5.64	5.76	4.23	4.68		5.83	4.47	3.77	
**Divorced**	7.93	8.38	7.3	7.3	8.58		8.31	7.71	7.06	
**Widowed/widower**	10.43	11.84	10.78	10.61	7.52		10.54	10.72	9.87	
**Maximum attained educational level (%)**						**<0.001**				NS
**Primary school or less**	48.59	57.14	48.56	45.19	38.37		49.73	47.88	45.99	
**Secondary school**	29.2	26.92	29.6	30.23	31.35		28.48	30.53	30.02	
**College or higher**	22.21	15.94	21.84	24.59	30.28		21.78	21.59	23.99	
**Schooling time (years)**	11.86 ± 8.51	11.35 ± 10.56	11.87 ± 7.98	11.97 ± 7.16	12.57 ± 6.33	**<0.001**	11.85 ± 9.2	11.75 ± 8.15	11.98 ± 6.55	NS
**Mean sleep hours at working days**	6.98 ± 1.22	6.96 ± 1.29	7.02 ± 1.2	6.95 ± 1.2	7.02 ± 1.16	NS	6.96 ± 1.25	6.96 ± 1.17	7.07 ± 1.18	**0.014**
**Mean sleep hours at weekend**	7.18 ± 1.28	7.12 ± 1.36	7.22 ± 1.24	7.16 ± 1.26	7.24 ± 1.22	**0.028**	7.17 ± 1.32	7.14 ± 1.23	7.22 ± 1.21	NS
**Labor status (%)**						**<0.001**				
**Active**	20.99	15.43	20.76	22.87	28.3		21.54	20.66	19.74	**<0.001**
**Long-term inactive**	23.01	28.89	22.57	21.09	15.96		24.75	21.36	19.6	
**Retired**	55.99	55.68	56.66	56.04	55.74		53.72	57.98	60.66	
**Diabetes at baseline n (%)**	1871 (27.22)	738 (31.54)	422 (28.27)	403 (24.71)	308 (21.84)	**<0.001**	1175 (28.53)	344 (26.52)	352 (24.13)	**0.004**
**Depression at baseline n (%)**	1428 (20.77)	627 (26.79)	282 (18.89)	278 (17.04)	241 (17.09)	**<0.001**	925 (22.46)	239 (18.43)	264 (18.09)	**<0.001**
**Hypertension at baseline n (%)**	5750 (83.65)	1994 (85.21)	1253 (83.92)	1360 (83.38)	1143 (81.06)	**0.01**	3467 (84.19)	1076 (82.96)	1207 (82.73)	NS
**History of lung disease n (%)**	23 (0.33)	7 (0.3)	9 (0.6)	2 (0.12)	5 (0.35)	NS	13 (0.32)	6 (0.46)	4 (0.27)	NS
**History of cancer n (%)**	497 (7.23)	185 (7.91)	103 (6.9)	117 (7.17)	92 (6.52)	NS	310 (7.53)	90 (6.94)	97 (6.65)	NS

Light = light-moderate PA activities but not every week; Moderate = moderate PA activities ≤150 min/week or vigorous PA activities ≤75 min/week; High = >150 min/week of moderate PA activities or >75 min/week of vigorous activities.

NS = non significant.

Q1 = 0–11 repetitions; Q2 = 12–13 repetitions; Q3 = 14–16 repetitions; Q4 = 17–37 repetitions.

Mean ± SD for age, weight, BMI, adherence to er-MedDiet, schooling time and sleep hours.

(%) for sex, smoking status, marital status, educational level, labor status and T2D, depression, hypertension, lung disease and cancer prevalence.

Chair stand test quartiles and PA levels were compared with sex, smoking status, marital status, educational level, labor status and T2D, depression, hypertension, lung disease and cancer prevalence with a χ^2^ test and with age, weight, BMI, adherence to er-MedDiet, schooling time and sleep hours with ANOVA test.

Significant p values in bold.

We found an association between the language domain of the MMSE test and the total MMSE score and PF (p = 0.024 in both cases) (Table 2). Both were higher in the upper PF quartile, although the increase in the score was below 1%. However, when we considered the PF score as a continuous variable, we lost significance after complete adjustment (Supplementary Table S1). We also found a higher calculation score associated with higher PF, although significance was lost after complete adjustment. Similar results were found with the PF score as a continuous variable (Supplementary Table S1). We found that better PF was associated with a higher score in phonemic (4.5%, 5.57% and 7.54% higher score for Q2, Q3 and Q4 *vs*. Q1, respectively) and semantic (3.24%, 5.86% and 6.53% higher score for Q2, Q3 and Q4 *vs*. Q1, respectively) verbal fluency tests (p < 0.001) (Table 2). Results were confirmed when we treated PF as a continuous variable (β = 0.0434 (0.0228–0.064) p = 0.001 for phonemic and 0.0428 (0.0203–0.0653) p = 0.003 for semantic fluency tests respectively) (Supplementary Table S1). TMT test time performance was also lower in higher PF quartiles. Specifically, TMT A time was 5.94%, 9.25%, and 10.32% lower and TMT B time was 6.14%, 10.04% and 11.05% lower in Q2, Q3 and Q4, respectively (p < 0.001 in both cases). When we analyzed PF as a continuous variable, we confirmed the lower TMT A time with higher PF score (β = −0.23 (−0.36–0.094), p = 0.0012), but we did not confirm the association between PF score and TMT B time (Supplementary Table S1). As sensitivity analyses, we excluded those with very high values of chair test (>2 SD, n = 237) as may represent implausible values/outliers and we lost the significant association with the language domain but not with total MMSE test score (p = 0.006), while association with TMT A and phonemic and semantic fluency tests remained significant, either analyzing PF quartiles or continuous scores (data not shown).

**Table 2:** Comparison of the cognitive assessment variables of the participants according to their physical fitness (quartiles).

		n		Q1 (n = 2340)	Q2 (n = 1493)	Q3 (n = 1631)	Q4 (n = 1410)	p value
**MMSE test**	**Temporal orientation**	6669	Model 1	4.84 (4.83–4.86)	4.87 (4.85–4.9)	4.88 (4.86–4.9)	4.88 (4.86–4.9)	NS
			Model 2	4.89 (4.8–4.98)	4.91 (4.82–5)	4.91 (4.82–5)	4.9 (4.81–4.99)	NS
	**Place orientation**	6669	Model 1	4.96 (4.95–4.97)	4.97 (4.95–4.98)	4.96 (4.95–4.97)	4.96 (4.95–4.98)	NS
			Model 2	4.94 (4.89–4.99)	4.94 (4.89–5)	4.94 (4.88–4.99)	4.94 (4.89–4.99)	NS
	**Immediate recall**	6670	Model 1	2.99 (2.98–3)	2.98 (2.97–2.99)	2.99 (2.98–3)	2.99 (2.98–3)	NS
			Model 2	3 (2.96–3.04)	3 (2.96–3.04)	3.01 (2.97–3.05)	3 (2.96–3.04)	NS
	**Calculation**	6670	Model 1	4.43 (4.39–4.48)	4.49 (4.43–4.54)	4.54 (4.49–4.59)	4.56 (4.5–4.62)	**0.038**
			Model 2	4.47 (4.24–4.69)	4.49 (4.27–4.71)	4.54 (4.31–4.76)	4.54 (4.32–4.77)	NS
	**Delay recall**	6670	Model 1	2.2 (2.16–2.24)	2.23 (2.19–2.28)	2.24 (2.2–2.28)	2.27 (2.22–2.32)	NS
			Model 2	2.22 (2.03–2.41)	2.26 (2.07–2.45)	2.27 (2.08–2.46)	2.27 (2.07–2.46)	NS
	**Language**	6650	Model 1	8.63 (8.61–8.66)	8.68 (8.65–8.71)	8.7 (8.67–8.73)	8.7 (8.66–8.73)	**0.038**
			Model 2	8.66 (8.53–8.79)	8.7 (8.57–8.83)	8.72 (8.59–8.85)	8.73 (8.59–8.86)	**0.024**
	**Total MMSE score**	6650	Model 1	28.06 (27.98–28.14)	28.23 (28.13–28.32)	28.32 (28.22–28.41)	28.35 (28.25–28.46)	**<0.001**
			Model 2	28.19 (27.79–28.58)	28.31 (27.91–28.71)	28.39 (27.99–28.79)	28.38 (27.98–28.79)	**0.024**
**CDT score**		6652	Model 1	5.87 (5.82–5.92)	5.96 (5.9–6.02)	5.93 (5.87–5.99)	6.02 (5.95–6.08)	NS
			Model 2	5.98 (5.71–6.24)	6.04 (5.77–6.31)	6.03 (5.76–6.29)	6.11 (5.84–6.38)	NS
**TMT test**	**Total time A**	6703	Model 1	55.87 (54.72–57.02)	52.73 (51.36–54.1)	51.27 (49.96–52.58)	50.3 (48.82–51.77)	**<0.001**
			Model 2	50.48 (44.74–56.21)	47.48 (41.72–53.24)	45.81 (40.01–51.61)	45.27 (39.46–51.08)	**<0.001**
	**Total time B**	6784	Model 1	143.33 (140.03–146.62)	135.91 (131.98–139.84)	130.38 (126.61–134.14)	127.29 (123.06–131.53)	**<0.001**
			Model 2	131.88 (115.33–148.44)	123.78 (107.15–140.4)	118.64 (101.9–135.38)	117.3 (100.53–134.07)	**<0.001**
**COWAT test**	**Phonemic fluency**	6723	Model 1	11.69 (11.5–11.87)	12.32 (12.1–12.55)	12.57 (12.35–12.78)	12.93 (12.69–13.17)	**<0.001**
			Model 2	12.21 (11.3–13.11)	12.76 (11.86–13.67)	12.89 (11.98–13.81)	13.13 (12.21–14.05)	**<0.001**
	**Semantic fluency**	6723	Model 1	15.54 (15.34–15.73)	16.05 (15.81–16.28)	16.5 (16.27–16.72)	16.62 (16.37–16.88)	**<0.001**
			Model 2	16.38 (15.39–17.37)	16.91 (15.91–17.9)	17.34 (16.34–18.34)	17.45 (16.45–18.46)	**<0.001**
**DS test**	**Direct numeric series score**	5345	Model 1	8.59 (8.47–8.7)	8.83 (8.7–8.97)	8.8 (8.67–8.93)	8.87 (8.72–9.01)	NS
			Model 2	8.56 (7.95–9.17)	8.68 (8.07–9.3)	8.54 (7.92–9.16)	8.51 (7.89–9.13)	NS
	**Direct span**	2213	Model 1	5.45 (5.33–5.56)	5.48 (5.34–5.62)	5.5 (5.37–5.63)	5.62 (5.46–5.77)	NS
			Model 2	5.58 (5.12–6.05)	5.62 (5.16–6.08)	5.58 (5.11–6.05)	5.68 (5.2–6.15)	NS
	**Inverse numeric series score**	5347	Model 1	4.96 (4.86–5.07)	5.15 (5.03–5.27)	5.11 (5–5.23)	5.19 (5.06–5.32)	NS
			Model 2	4.83 (4.3–5.36)	4.88 (4.34–5.42)	4.74 (4.2–5.28)	4.74 (4.2–5.28)	NS
	**Inverse Span**	2200	Model 1	3.6 (3.49–3.7)	3.73 (3.61–3.86)	3.77 (3.65–3.89)	3.81 (3.67–3.95)	NS
			Model 2	3.69 (3.28–4.09)	3.81 (3.41–4.21)	3.78 (3.37–4.19)	3.83 (3.42–4.24)	NS

COWAT = Controlled Oral Word Association Test; DS = Digit Span test; MMSE = Mini-mental State Examination test; TMT = Trail Making Test.

NS = non significant.

Q1 (0–11 repetitions); Q2 (12–13 repetitions); Q3 (14–16 repetitions); Q4 (17–37 repetitions).

Mean ± (95% confidence interval).

Model 1: ANCOVA adjusted for age, sex and recruitment center.

Model 2: ANCOVA adjusted for age, sex, recruitment center, smoking status, adherence to er-MedDiet, BMI, civil status, educational level, sleep hours, working status, TV hours and prevalence of type 2 diabetes, depression, hypertension and cancer.

Significant p values in bold.

### Association between PA levels and energy expenditure and cognitive function

Participants with the highest level of PA were mainly men (p < 0.001), former smokers (p < 0.001), with high adherence to MedDiet (p < 0.001), slept longer time in weekdays (p = 0.014) and showed lower weight (p < 0.001) and BMI (p < 0.001) (Table 1). Married participants were more frequently classified as high PA level, whereas single, divorced or widow participants were more frequently classified as light PA level (p = 0.018). Additionally, current workers showed a lower PA level than retired participants (p < 0.001). Finally, the prevalence of diabetes and depression was lower among participants with a high PA level (p = 0.017 and p = 003, respectively) (Table 1). We found a positive association between higher PA and higher scores in phonemic (2.39% and 5.45% for moderate and high PA level vs. low PA level, p < 0.001) and semantic fluency tests (0.25% and 3.88% for moderate and high PA level vs. low PA level, p = 0.003) under the minimally adjusted model (Table 3). However, the significance was lost after complete adjustment. We did not find any association between the level of PA and the score in any of the neurocognitive tests in the complete adjustment model (Table 3). Similarly, we found higher scores in the direct numeric series associated with higher PA level under the minimally adjusted model (1.73% and 3.46% for moderate and high PA levels compared with low PA level, p = 0.032), but significance was lost after complete adjustment (Table 3). Similar results were observed when analyzing the association between scoring in neurocognitive tests and energy expenditure with total PA (Supplementary Table S1).

**Table 3:** Comparison of the cognitive assessment variables of the participants according to their PA level.

		n		Low (n = 4118)	Moderate (n = 1297)	High (n = 1459)	p for trend
**MMSE test**	**Temporal orientation**	6669	Model 1	4.86 (4.85–4.87)	4.88 (4.86–4.91)	4.87 (4.85–4.9)	NS
			Model 2	4.9 (4.81–4.99)	4.92 (4.83–5.01)	4.88 (4.79–4.98)	NS
	**Place orientation**	6669	Model 1	4.96 (4.95–4.97)	4.96 (4.94–4.97)	4.96 (4.95–4.98)	NS
			Model 2	4.94 (4.89–4.99)	4.94 (4.89–4.99)	4.93 (4.88–4.98)	NS
	**Immediate recall**	6670	Model 1	2.99 (2.98–2.99)	2.98 (2.97–2.99)	2.99 (2.98–3)	NS
			Model 2	3 (2.96–3.04)	3 (2.96–3.04)	3 (2.96–3.04)	NS
	**Calculation**	6670	Model 1	4.49 (4.45–4.52)	4.5 (4.44–4.56)	4.53 (4.47–4.59)	NS
			Model 2	4.51 (4.28–4.73)	4.49 (4.26–4.71)	4.49 (4.26–4.72)	NS
	**Delayed recall**	6670	Model 1	2.23 (2.2–2.26)	2.22 (2.18–2.27)	2.23 (2.18–2.28)	NS
			Model 2	2.25 (2.06–2.44)	2.25 (2.05–2.44)	2.24 (2.05–2.44)	NS
	**Language**	6650	Model 1	8.67 (8.65–8.69)	8.66 (8.62–8.69)	8.68 (8.64–8.71)	NS
			Model 2	8.7 (8.57–8.83)	8.68 (8.55–8.81)	8.7 (8.56–8.83)	NS
	**Total MMSE score**	6650	Model 1	28.2 (28.14–28.26)	28.21 (28.11–28.32)	28.27 (28.16–28.37)	NS
			Model 2	28.31 (27.92–28.7)	28.28 (27.88–28.68)	28.25 (27.85–28.65)	NS
**CDT score**		6652	Model 1	5.95 (5.91–5.99)	5.93 (5.86–6)	5.9 (5.83–5.97)	NS
			Model 2	6.03 (5.77–6.3)	6.03 (5.76–6.3)	6.01 (5.74–6.28)	NS
**TMT test**	**Total time A**	6703	Model 1	53.5 (52.65–54.35)	52.66 (51.19–54.13)	51.41 (49.91–52.92)	NS
			Model 2	48.28 (42.58–53.98)	47.97 (42.16–53.79)	45.82 (39.99–51.65)	NS
	**Total time B**	6784	Model 1	136.59 (134.15–139.03)	133.04 (128.81–137.26)	133.48 (129.16–137.81)	NS
			Model 2	123.82 (107.36–140.28)	126.1 (109.31–142.89)	125.46 (108.63–142.3)	NS
**COWAT test**	**Phonetic fluency**	6723	Model 1	12.11 (11.97–12.25)	12.4 (12.16–12.64)	12.77 (12.53–13.02)	**<0.001**
			Model 2	12.6 (11.7–13.5)	12.67 (11.75–13.59)	12.89 (11.97–13.81)	NS
	**Semantic fluency**	6723	Model 1	15.97 (15.83–16.12)	16.01 (15.75–16.26)	16.59 (16.33–16.85)	**0.003**
			Model 2	16.88 (15.9–17.87)	16.69 (15.69–17.7)	17.21 (16.21–18.22)	NS
**DS test**	**Direct numeric series score**	5345	Model 1	8.67 (8.59–8.75)	8.82 (8.67–8.97)	8.97 (8.82–9.12)	**0.032**
			Model 2	8.58 (7.98–9.19)	8.55 (7.93–9.17)	8.55 (7.93–9.17)	NS
	**Direct span**	2213	Model 1	5.47 (5.36–5.57)	5.51 (5.38–5.65)	5.54 (5.4–5.68)	NS
			Model 2	5.61 (5.15–6.07)	5.6 (5.13–6.06)	5.64 (5.18–6.11)	NS
	**Inverse numeric series score**	5347	Model 1	5.05 (4.97–5.12)	5.14 (5–5.27)	5.18 (5.05–5.32)	NS
			Model 2	4.82 (4.29–5.35)	4.82 (4.28–5.36)	4.72 (4.18–5.26)	NS
	**Inverse Span**	2200	Model 1	3.68 (3.59–3.78)	3.67 (3.55–3.79)	3.78 (3.65–3.91)	NS
			Model 2	3.76 (3.36–4.16)	3.74 (3.33–4.14)	3.85 (3.44–4.26)	NS

COWAT = Controlled Oral Word Association Test; DS = Digit Span test; MMSE = Mini-mental State Examination test; TMT = Trail Making Test.

NS = non significant.

Light = light-moderate PA activities but not every week; Moderate = moderate PA activities ≤150 min/week or vigorous PA activities ≤75 min/week; High = >150 min/week of moderate PA activities or >75 min/week of vigorous activities.

Mean ± (95% confidence interval).

Model 1: ANCOVA adjusted for age, sex and recruitment center.

Model 2: ANCOVA adjusted for age, sex, recruitment center, smoking status, adherence to er-MedDiet, BMI, civil status, educational level, sleep hours, working status, TV hours and prevalence of type 2 diabetes, depression, hypertension and cancer.

Significant p values in bold.

### Association between both, PF and PA and health-related quality of life

Higher levels of PA and higher energy expenditure with total PA were associated with higher scores in all domains of the SF36-HRQL questionnaire (Table 4 and Supplementary Table S2). Accordingly, individuals in the upper quartile of PF or with higher Chair Stand repetitions showed higher scores in all domains of the HRQL-SF36 questionnaire, including the aggregated physical and mental health domains (Table 5 and Supplementary Table S2). We repeated the analysis excluding those with very high values of chair test (>2 SD, n = 237) and results did not change (data not shown).

**Table 4:** Adjusted means for HRQL dimensions according to baseline PA levels.

	**n**		Low (n = 4118)	Moderate (n = 1297)	High (n = 1459)	p value
**General health**	6490	Model 1	58.58 (58.01–59.16)	62.9 (61.91–63.9)	65.63 (64.61–66.65)	**<0.001**
		Model 2	50.57 (46.85–54.28)	53.67 (49.89–57.46)	55.33 (51.53–59.13)	**<0.001**
**Physical function**	6363	Model 1	72.81 (72.22–73.4)	79.35 (78.34–80.37)	83.07 (82.03–84.1)	**<0.001**
		Model 2	67.19 (63.29–71.1)	72.18 (68.2–76.17)	74.51 (70.52–78.5)	**<0.001**
**Physical role**	6636	Model 1	72.15 (71.04–73.27)	78.37 (76.44–80.31)	81.94 (79.96–83.92)	**<0.001**
		Model 2	55.88 (48.52–63.24)	60.54 (53.03–68.05)	63.05 (55.52–70.58)	**<0.001**
**Emotional role**	6678	Model 1	85 (84.03–85.96)	89.17 (87.51–90.83)	89.48 (87.78–91.18)	**<0.001**
		Model 2	77.94 (71.34–84.53)	80.75 (74.02–87.48)	80.58 (73.83–87.33)	**0.002**
**Vitality**	6435	Model 1	60.32 (59.66–60.99)	65.85 (64.7–67)	69.18 (68–70.36)	**<0.001**
		Model 2	56.77 (52.47–61.07)	60.7 (56.31–65.08)	63.14 (58.74–67.54)	**<0.001**
**Mental health**	6269	Model 1	72.84 (72.22–73.46)	75.7 (74.62–76.78)	77.31 (76.22–78.41)	**<0.001**
		Model 2	67.05 (63.17–70.94)	68.71 (64.74–72.68)	69.95 (65.97–73.93)	**<0.001**
**Social function**	6591	Model 1	83.44 (82.77–84.1)	88.22 (87.07–89.37)	89.71 (88.53–90.88)	**<0.001**
		Model 2	75.9 (71.46–80.33)	79.48 (74.96–84)	80.38 (75.84–84.92)	**<0.001**
**Body pain**	6719	Model 1	64.44 (63.63–65.25)	69.1 (67.7–70.5)	73.81 (72.38–75.25)	**<0.001**
		Model 2	53.91 (48.48–59.33)	57.11 (51.58–62.64)	60.69 (55.14–66.23)	**<0.001**
**Aggregated Physical Dimensions**	5446	Model 1	44.17 (43.87–44.47)	46.21 (45.7–46.73)	48.09 (47.57–48.61)	**<0.001**
		Model 2	40.66 (38.67–42.66)	42.13 (40.09–44.17)	43.5 (41.45–45.54)	**<0.001**
**Aggregated Mental Dimensions**	5446	Model 1	50.5 (50.14–50.86)	51.76 (51.13–52.39)	51.96 (51.32–52.59)	**<0.001**
		Model 2	48.25 (45.83–50.67)	49.05 (46.58–51.52)	49.12 (46.64–51.59)	**0.011**

Light = light-moderate PA activities but not every week; Moderate = moderate PA activities ≤150 min/week or vigorous PA activities ≤75 min/week; High = >150 min/week of moderate PA activities or >75 min/week of vigorous activities.

Mean ± (95% confidence interval).

Model 1: ANCOVA adjusted for age, sex and recruitment center.

Model 2: ANCOVA adjusted for age, sex, recruitment center, smoking status, adherence to er-MD, BMI, civil status, educational level, sleep hours, working status, TV hours and prevalence of type 2 diabetes, depression, hypertension and cancer.

Significant p values in bold.

**Table 5:** Adjusted means for HRQL dimensions according to the baseline level of PF (quartiles).

	**n**		Q1 (n = 2340)	Q2 (n = 1493)	Q3 (n = 1631)	Q4 (n = 1410)	p value
**General health**	6490	Model 1	56.53 (55.75–57.3)	61.42 (60.49–62.35)	63.13 (62.24–64.02)	64.29 (63.29–65.28)	**<0.001**
		Model 2	49.15 (45.4–52.89)	52.94 (49.18–56.7)	53.7 (49.91–57.49)	54.08 (50.29–57.87)	**<0.001**
**Physical function**	6363	Model 1	69.04 (68.25–69.83)	76.87 (75.94–77.81)	79.67 (78.76–80.57)	82.3 (81.3–83.3)	**<0.001**
		Model 2	64.18 (60.27–68.09)	70.85 (66.93–74.77)	72.42 (68.47–76.36)	73.61 (69.65–77.56)	**<0.001**
**Physical role**	6636	Model 1	66.9 (65.39–68.4)	76.55 (74.76–78.34)	80.23 (78.51–81.94)	81.6 (79.68–83.52)	**<0.001**
		Model 2	51.56 (44.17–58.94)	59.87 (52.46–67.28)	62.53 (55.06–69.99)	62.79 (55.32–70.27)	**<0.001**
**Emotional role**	6678	Model 1	83.04 (81.74–84.35)	88.2 (86.64–89.76)	88.13 (86.64–89.62)	89.23 (87.57–90.9)	**<0.001**
		Model 2	76.01 (69.37–82.65)	80.23 (73.55–86.91)	80.2 (73.49–86.92)	81.17 (74.45–87.9)	**<0.001**
**Vitality**	6435	Model 1	57.62 (56.72–58.51)	63.52 (62.44–64.59)	66.02 (65–67.05)	68.12 (66.98–69.27)	**<0.001**
		Model 2	54.67 (50.34–58.99)	59.28 (54.94–63.62)	61.26 (56.89–65.64)	62.48 (58.09–66.86)	**<0.001**
**Mental health**	6269	Model 1	70.86 (70.03–71.69)	75.35 (74.35–76.34)	75.88 (74.93–76.84)	76.77 (75.7–77.84)	**<0.001**
		Model 2	65.22 (61.31–69.13)	68.73 (64.81–72.66)	69.37 (65.41–73.32)	69.96 (66–73.93)	**<0.001**
**Social function**	6591	Model 1	81.01 (80.11–81.9)	86.88 (85.81–87.96)	87.93 (86.9–88.95)	88.86 (87.71–90)	**<0.001**
		Model 2	73.73 (69.27–78.19)	78.8 (74.32–83.28)	79.42 (74.92–83.93)	79.82 (75.3–84.33)	**<0.001**
**Body pain**	6719	Model 1	60.02 (58.94–61.11)	68.13 (66.83–69.42)	71.11 (69.86–72.35)	73.1 (71.7–74.49)	**<0.001**
		Model 2	50.47 (45.02–55.91)	57.08 (51.62–62.54)	58.93 (53.43–64.43)	59.62 (54.11–65.13)	**<0.001**
**Aggregated Physical Dimensions**	5446	Model 1	42.47 (42.07–42.87)	45.69 (45.22–46.17)	46.85 (46.39–47.3)	47.81 (47.31–48.31)	**<0.001**
		Model 2	39.34 (37.34–41.34)	42.04 (40.03–44.06)	42.58 (40.56–44.6)	42.91 (40.88–44.93)	**<0.001**
**Aggregated Mental Dimensions**	5446	Model 1	49.91 (49.42–50.4)	51.55 (50.96–52.14)	51.62 (51.05–52.18)	51.61 (50.99–52.23)	**<0.001**
		Model 2	47.59 (45.15–50.02)	48.88 (46.43–51.33)	49.15 (46.69–51.61)	49.18 (46.72–51.64)	**<0.001**

Q1 (0–11 repetitions); Q2 (12–13 repetitions); Q3 (14–16 repetitions); Q4 (17–37 repetitions).

Mean ± (95% confidence interval).

Model 1: ANCOVA adjusted for age, sex and recruitment center.

Model 2. ANCOVA adjusted for age, sex, recruitment center, smoking status, adherence to er-MD, BMI, civil status, educational level, sleep hours, working status, TV hours and prevalence of type 2 diabetes, depression, hypertension and cancer.

Significant p values in bold.

## Discussion

Our study showed that PF was positively associated with a better performance in the TMT, measuring executive function, and the COWAT, measuring language ability and executive control. These results are in accordance with those reported by Alosco *et al*. in 200 adult patients of heart failure using a similar battery of cognitive and PF tests^28^. The authors suggested that the negative effect of obesity on cognitive function was likely a result of the combination of clinical factors (obesity, diabetes, and hypertension) and PF^28^. Because we included BMI, diabetes, and hypertension as adjusting co-variables, our results suggest that PF influences cognitive function independently of these other factors.

We did not find any association between the level of PA and cognitive function. The association between PA and cognitive function is still controversial. Some previous reports have suggested a positive association. For instance, a study conducted in 2,315 cognitively healthy elder participants from CFAS-Wales study showed that PA was positively associated with cognitive function measured by the Cambridge Cognitive Examination battery of neuropsychological tests^29^ and a larger observational study carried out in 93,082 respondents ≥45 years old from 21 US states reported that people with self-reported cognitive decline were more frequently inactive than those without this subjective cognitive decline status^30^. Moreover, the study conducted by Xu *et al*. in 27,651 participants (50–85 years old) showed a dose-response relationship between PA and cognitive function^14^. Other population studies and meta-analyses support an association between PA and cognitive function^20,22,31^. Conversely, Kooistra *et al*. did not find any association between PA and cognitive performance or cognitive decline in a baseline analysis of 1,232 participants of the SMART-RM study^16^. These inconsistencies could be related to differences in populations and tools used to measure cognitive function and PA. The intensity of the PA could also be a determinant factor. The OSHPE study found that moderate activity, but not light activity, was associated with hippocampal volume but not with memory^15^ and a recent systematic review concluded that moderate and vigorous leisure-time PA was associated with benefits in different cognitive domains in older adults^17^. Therefore, it is possible that an association between PA and cognitive function was significant only in individuals at a high PA level. Indeed, our results show a trend towards a better scoring in TMT and COWAT test in participants categorized as high-level PA.

The discordant results could also be attributed to the type of PA, aerobic, or resistance/strength exercises, with aerobic exercises likely having a greater effect. Protocols for each type of exercise are different in intensity and length and produce different physical adaptations, with aerobic exercise having a greater impact on %VO_2_max and energy expenditure^32–34^. We found that, although higher PA and PF levels were both associated with better self-reported quality of life, only PF was associated with cognitive function. This differential effect could be attributed to a different impact of aerobic vs. anaerobic exercise on cognitive function. Aerobic fitness has been positively correlated with better high-interference memory in older adults^18^, and aerobic exercises have been associated with better improvement in the score of neuropsychological tests in comparison with anaerobic exercise^35^. Aerobic exercise in the elderly has also been associated with better performance on tasks requiring executive control in comparison with anaerobic exercises^36^. Other reports also showed a specific association between aerobic exercise, but not anaerobic exercise, and executive control^37,38^.

Moreover, a meta-analysis published in 2003 found robust, but specific, benefits of aerobic fitness on cognitive function^19^. Another meta-analysis including 29 randomized interventional trials involving aerobic exercise training (n = 2049) found a moderate but consistent improvement in attention and processing speed, executive function and memory associated with aerobic exercise. However, it failed to find any association with working memory^21^. These studies strongly support the hypothesis that aerobic exercise, but not anaerobic one, has a specific impact on executive function. We did not discriminate between aerobic and anaerobic exercise, neither measured cardiorespiratory fitness; however, we found a significant association between TMT and COWAT tests and PF. TMT test measures the speed of information processing and the COWAT test measures verbal memory, attention, and inhibition. Together, they define the individual condition regarding executive function and cognitive control. Therefore, our results strongly suggest that good PF, achieved through an aerobic exercise program, could be a significant predictor of executive function in the elderly. The mechanisms behind these associations could be the increase in the hippocampal volume associated with aerobic fitness^39^. In this regard, a randomized clinical trial conducted in 120 adults without dementia showed an increase in the volume of the left and right hippocampus in the aerobic exercise group and a decrease in the stretching control group after one year^34^.

Interestingly, authors showed a selective impact of aerobic exercise on the volume of the anterior hippocampus, involved in spatial memory and submitted to age-related atrophy^34^. However, a Cochrane systematic review showed no effect of aerobic exercise, even if the intervention leads to an improvement in cardiorespiratory fitness, on cognitive domains^23^. More extensive interventional studies are, therefore, needed to clarify the impact of aerobic exercise leading to an improved PF on cognitive function.

We also found that PF was associated with a better quality of life, with positive associations in all domains, including the aggregated ones. PF has been reported to account for up to 22.5% of the variability in the quality of life scoring of obese individuals with binge eating disorder^40^, and cardiorespiratory fitness has been suggested to be a good predictor of physical and mental quality of life in individuals with bipolar disorder^41^. Our results reinforce the hypothesis that PF is associated with a better cognitive function and quality of life and encourage the development of PA programs addressed to increase PF in the late adulthood to prevent morbidity in the elderly. Although PA levels seemed not to be associated with cognitive function, they were associated with a higher score in all domains of the quality of life questionnaire. These results are consistent with the previously reported association between PA and quality of life in the elderly^42,43^.

In conclusion, our results showed that PF, but not PA level, was associated with cognitive function, specifically with those domains related to language ability, attention, and processing speed. Undoubtedly, PA, specifically aerobic exercises, contribute to PF and thus PA could be associated with cognitive function through an improvement in PF. Our results highlight the importance of maintaining a good physical condition to retain cognitive function. In this regard, the primary aim of PA programs addressed to maintain cognitive health should be the improvement of PF, and the type of exercise should be considered when designing PA programs.

This study has some limitations. First, this is an observational cross-sectional study that does not allow to infer causality. Second, this is an elderly population with obesity and metabolic syndrome with an average low level of PA and without cognitive impairment and this could be masking a significant relationship between PA and cognitive function. However, this population is representative of a high cardiovascualr disease (CVD) risk elderly population in a medium-high income Western country and this study has been carried out with a large sample and with a stringent adjustment for confounding factors. Third, HRQL and PA activities are self-reported; thus, we cannot rule out the overestimation or underestimation of those variables. Although we have adjusted for potential confounding factors (age, gender, recruitment center, adherence to an er-MedDiet, BMI, civil status, educational level, sleep hours, working status, TV hours, prevalence of T2DM, depression, hypertension and cancer) our population is heterogeneous, especially regarding sociodemographic parameters like civil status, education level and working status. Thus, we cannot rule out the possibility that this heterogeneity produced a moderation or exacerbation of the observed association between PF and cognitive function.

Finally, we should bear in mind that past experiences or the accumulation of other factors, not considered within this analysis, may also have an impact on cognitive function in the elderly.

## Methods

### Study design and participants

We used baseline data of the PREDIMED-Plus trial, a 6-year multicenter, randomized, parallel-group clinical trial conducted in Spain for primary prevention of CVD through lifestyle modification. The cohort and protocol, including sample size calculation and recruitment flowchart, have been described^27^, and the protocol can be found at the PREDIMED-Plus website^44^. This trial was retrospectively registered at the International Standard Randomized Controlled Trial (ISRCTN89898870, https://www.isrctn.com/ISRCTN89898870?q=ISRCTN89898870&filters=&sort=&offset=1&totalResults=1&page=1&pageSize=10&searchType=basic-search) on 07/24/2014. Participants were recruited by primary care health doctors and nurses associated with study centers who assessed the inclusion/exclusion criteria. Eligible participants were community-dwelling adults (55–75 years men and 60–75 years women) with overweight/obesity (27 ≤ BMI ≥ 40 Kg/m^2^) who met at least three criteria of metabolic syndrome (according to the International Diabetes Federation and the American Heart Association)^45^ without cardiovascular and neurodegenerative disease at baseline. We included baseline data from 6874 participants recruited in 23 Spanish study centers from October 2013 to December 2016. Data include cognitive function, PA, quality of life, anthropometrical and biochemical measures, PF conditions, history of cancer or lung, disease sociodemographic parameters, and depression status^27^.

The study protocol and procedures were approved according to the ethical standards of the Declaration of Helsinki by the Institutional Review Boards (IRBs) of all the participating institutions: CEI Provincial de Málaga, CEI de los Hospitales Universitarios Virgen Macarena y Virgen del Rocío, CEI de la Universidad de Navarra, CEI de las Illes Balears, CEIC del Hospital Clínic de Barcelona, CEIC del Parc de Salut Mar, CEIC del Hospital Universitari Sant Joan de Reus, CEI del Hospital Universitario San Cecilio, CEIC de la Fundación Jiménez Díaz, CEIC Euskadi, CEI en Humanos de la Universidad de Valencia, CEIC del Hospital Universitario de Gran Canaria Doctor Negrín, CEIC del Hospital Universitario de Bellvitge, CEIC de IMDEA Alimentación, CEIC del Hospital Clínico San Carlos, CEI Provincial de Málaga, CCEIBA de la Investigación Biomédica de Andalucía, CEIC del Hospital General Universitario de Elche, Comité de Ética del Hospital Universitario Reina Sofía and CEIC de León. All participants provided informed written consent.

### Dietary and anthropometrical measures

Adherence to an er-MedDiet diet was measured through a 17-item questionnaire in which an adequate consumption of typical traditional Mediterranean foods adds add one point, and low consumption of foods that are not characteristic of the traditional Mediterranean diet also adds one point. This questionnaire has been specially developed for the PREDIMED-Plus trial^11^ and was applied by a trained nutritionist in a face-to-face interview. Weight and height were measured with light clothing and no shoes, with calibrated scales and a wall-mounted stadiometer, respectively. BMI was calculated as weight in kilograms divided by the square of height in meters.

### Physical activity and fitness

PF was assessed using the 30 seconds Chair Stand test designed to test the functional fitness of senior population^46^. Participants were classified according to quartiles of test scoring: Q1 (0–11 repetitions), Q2 (12–13 repetitions), Q3 (14–16 repetitions) and Q4 (17–37 repetitions).

PA was assessed using the validated REGICOR questionnaire and the Rapid Assessment of Physical Activity Questionnaire (RAPA)^47^. REGICOR questionnaire provides information about PA intensity according to energy expenditure (MET/min/week) distinguishing light (<4 MET), moderate (4–5.5 MET) and vigorous (≥6 MET) PA and recording information about the PA at work and in everyday life and about sedentary behaviors^42^. Frequency and intensity of PA are quantified by requesting participants to provide information about the frequency (days per month) and the intensity (minutes per session) of physical activities like light walking, brisk walking, gardening, trail/hiking, climbing stairs, other indoor or outdoor sports activities. RAPA questionnaire allows an easy identification of PA level as low (light-moderate PA activities but not every week), moderate (moderate PA activities less than 150 minutes per week or vigorous PA activities less than 75 minutes per week) or high (more than 150 minutes per week of moderate PA activities or more than 75 minutes per week of vigorous activities). Trained interviewers administered PA questionnaires in individual face-to-face sessions.

### Neuropsychological assessment

Executive function was measured with the Trail-making Test (TMT) A and B^3,48^. TMT-A provides information about cognitive processing skills (psychomotor speed, processing speed, and visuospatial skills), whereas TMT-B provides information about executive functioning^49^. In task A, participants are requested to connect circled numbers 1–25 in the correct order. In task B, numbers and letters have to be alternatively connected following an ascending order in each case (1-A-2-B-3-C-…).

Semantic and phonemic verbal ability and executive control were measured with the Controlled Oral Word Association Test (COWAT)^3,50^. Semantic fluency is evaluated by requesting participants to mention as many animals as possible in 1 minute. Phonemic fluency is evaluated by requesting participants to say as many words starting with “P” as they can in 1 minute^50^.

Short-term memory and attention and working memory were measured by the forward and backward Digit Span (DS) test of the Wechsler Adult Intelligence Scale-III battery^3,51^, respectively. In the DS-Forward recall, participants are requested to repeat a series of random single digits in the same order they heard them. In the DS-Backward recall they are requested to repeat another series of a random single digit in the inverse order they heard them.

Cognitive impairment was assessed with the Mini-mental State Examination test (MMSE) and the Clock-Drawing test (CDT)^52–54^. The MMSE test is a 30-point questionnaire that examines domains like time and spatial orientation, immediate and deferred recall, attention, calculation, and language. A score is calculated for each domain, and all are added to obtain a total MMSE score. A threshold of 24 is defined to identify cognitive impairment. The CDT is useful to examine visuospatial functioning and memory. Participants are required to draw a clock with a specific time, and a numeric score of 0 or 1 is given depending if they correctly or incorrectly draw the numbers, minute and seconds clock hands and the requested time^49^. All scores are sum to get a total CDT score.

All cognitive tests were applied by nutritionists who had been previously trained.

### Health-related quality of life (HRQL)

We used an adapted version of previously published 36-items HRQL questionnaire (SF36-HRQL)^55^, which was validated for the Spanish population and extensively used to measure the subjective perception of health and capability or physical limitations to manage with daily tasks^56^, as described before^11,57^.

### Statistical analyses

Baseline characteristics of the participants were described as means ± SD for quantitative variables and as proportions for qualitative variables. Chair Stand test scores and MET/Min/week of total PA were used to quantitatively measure PF and PA, respectively, and were analyzed as continuous variables. Additionally, participants were classified according to the quartile of PF measured by the Chair Stand test.: Q1 (0–11 repetitions), Q2 (12–13 repetitions), Q3 (14–16 repetitions) and Q4 (17–37 repetitions). PA activity level was also analyzed as a categorical variable: low, moderately, and highly active. The participant’s categorization has been described above.

Characteristics of the participants were compared among PF quartiles and PA levels with the χ^2^ test for categorical variables and ANOVA for continuous variables.

The score for each domain of the SF36-HRQL tests as well as for the aggregated dimensions was treated as a continuous variable and compared with PA and PF with ANCOVA. Neurocognitive scores were also treated as continuous variables and compared with PA levels and PF with ANCOVA.

SF36-HRQL and neurocognitive scores (independent variables) were analyzed for their association with Chair Stand test score and MET/min/week energy expenditure as continuous variables (dependent variables) with a linear regression model.

Two adjustment models were applied. Model 1 adjustment (minimal adjustment) included age, gender, and recruitment center as cofactors. Model 2 adjustment (complete adjustment) included, additionally, smoking status (current, never or former smoker), adherence to er-MedDiet^11^, BMI, civil status (married, single, divorced or widow), educational level (primary, secondary or college), sleep hours, working status (active, long-term inactive or retired), TV hours, prevalence of T2DM^3^, depression, hypertension and cancer. We included data from 6874 participants. Missing values for predictive and descriptive variables were imputed using Predictive Mean Matching. The specific sample size for outcome variables is detailed in corresponding tables. The percentage of missing values range from 0.029 to 9%, except for the DS test and the aggregated physical and mental dimensions of the SF36-HRQL test, for whose percentage of missing values ranged from 20.99% to 67.99%. To prevent from type-I error inflation, a multiple testing correction with the Holm’s method was applied^58^. Holm’s method is a sequential hypothesis rejection test that controls the family-wise error rate like Bonferroni’s one but with much increased power. As sensitivity analyses, we excluded participants with >2 SD values of the Chair Stand Test (n = 237) as may represent implausible values/outliers and repeated ANCOVA analyses. We considered the two-tailed significant level of 0.05 as a threshold for statistical significance. All analyses were conducted with data from database version 201903131142_PREDIMEDplus_2019-03-12 and with R.

## Supplementary information

List of additional PREDIMED-Plus investigators.

Supplementary Table 1.

Supplementary Table 2.

## Data Availability

The use of the dataset supporting the conclusions of this article is restricted due to the signed consent agreements around data sharing, which only allow access to external researchers for studies following the project purposes. Requestors wishing to access the PREDIMED-Plus trial data used in this study can make a request to the PREDIMED-Plus trial Steering Committee chair: jordi.salas@urv.cat. The request will then be passed to members of the PREDIMED-Plus Steering Committee for deliberation.
