# Supplementary material for: Physical fitness and physical activity association with cognitive function and quality of life: baseline cross-sectional analysis of the PREDIMED-Plus trial
Source: Sci Rep. 2020 Feb 26;10:3472. doi: 10.1038/s41598-020-59458-6 (PMC7044289; doi:10.1038/s41598-020-59458-6)
Supplement: Supplementary file 1 — List of additional PREDIMED-Plus investigators. [file 41598_2020_59458_MOESM1_ESM.pdf]

Physical fitness and physical activity association with cognitive function and quality of life: baseline cross-sectional analysis of the PREDIMED-Plus trial

Lidia Daimiel, PhD[1*], Miguel A Martínez-González, MD, PhD[2], Dolores Corella, PharmD, PhD[3], Jordi Salas-Salvadó, MD, PhD[4], Helmut Schröder, PhD[5], Jesús Vioque, MD, PhD[6], Dora Romaguera, PhD[7], José A Martínez, MD, PhD[8], Julia Wärnberg, PhD[9], Jose Lopez-Miranda, MD, PhD[10], Ramón Estruch, MD, PhD[11], Naomi Cano-Ibáñez, MD[12], Angel Alonso-Gómez, MD, PhD[13], Josep A Tur, PhD[14], Francisco J Tinahones, MD, PhD[15], Lluis Serra-Majem MD, PhD[16], Rafael M Micó-Pérez, MD, PhD[17], José Lapetra, MD, PhD[18], Alba Galdón, MD[19], Xavier Pintó, MD, PhD[20], Josep Vidal, MD, PhD[21], Victor Micó, PhD[1], Gonzalo Colmenarejo, PhD[22], José J Gaforio, MD, PhD[23], Pilar Matía, MD, PhD[24], Emilio Ros, MD, PhD[5], Pilar Buil-Cosiales, MD, PhD[25], Zenaida Vázquez, RD[26], José V Sorlí, MD, PhD[3], Indira Paz Graniel, MD[4], Aida Cuenca-Royo, PhD[27], Cristina Gisbert-Sellés, MD[6,28], Aina M Galmes-Panades, MSc[7], M Angeles Zulet, PhD[8], Antonio García-Ríos, MD, PhD[10], Andrés Díaz-López, RD, PhD[4], Rafael de la Torre, MD, PhD[5], Iñigo Galilea-Zabalza, MD[29], José M. Ordovás, PhD[1,30]

PREDIMED-PLUS INVESTIGATORS

Steering committee: D. Corella, R. Estruch, M. Fitó, M.A. Martínez-González, E. Ros, J. Salas-Salvadó (principal investigator), and F. Tinahones.

Clinical Event Ascertainment Committee: F. Arós, M. Aldamiz, A. Alonso, J. Berjón, L. Forga, J. Gállego, M.A. García Layana, A. Larrauri, J. Portu, J. Timiraos, M. Serrano-Martínez.

Dietary Intervention Committee: J. Salas-Salvadó, N. Babio, E. Ros, A. Sánchez-Tainta.

Physical Activity Committee: M. Fitó, H. Schröder, A. Marcos, D. Corella, J. Wärnberg.

Behaviour Treatment Committee: R. Estruch, F. Fernández-Aranda, C. Botella, J. Salas-Salvadó.

Independent Data and Safety Monitoring Board: M.J. Stampfer (Harvard School of Public Health), J. Sabate (Loma Linda University), A. Astrup (Copenhagen University), F. Fernandez-Aviles (Universidad Complutense of Madrid), X. Pi-Sunyer (Columbia University).

Department of Preventive Medicine and Public Health, University of Navarra-Navarra Institute for Health Research and Primary Care Centres, Pamplona, Spain: C. Razquin, M. Ruiz-Canela M. Bes-Rastrollo, A. Sanchez Tainta, B. SanJulian Aranguren, E. Goñi, I. Barrientos, M. Canales, A. Rico, J. Basterra Gortari, A. Garcia Arellano, O. Lecea-Juarez, J. Carlos Cenoz-Osinaga, J. Bartolome-Resano, A. Sola-Larraza, E. Lozano-Oloriz, B. Cano-Valles, S. Eguaras, E. Pascual Roquet-Jalmar, H. Lancova, R. Ramallal, M.L. Garcia-Perez, V. Estremera-Urabayen, M.J. Ariz-Arnedo, C. Hijos-Larraz, C. Fernandez Alfaro, B. Iñigo-Martinez, R. Villanueva Moreno, S. Martin-Almendros, L. Barandiaran-Bengoetxea, C. Fuertes-Goñi, A. Lezaun-Indurain, M.J. Guruchaga-Arcelus, O. Olmedo-Cruz, B. Iñigo-Martínez, L. Escriche-Erviti, R. Ansorena-Ros, R. Sanmatin-

Zabaleta, J. Apalategi-Lasa, J. Villanueva-Telleria, M.M. Hernández-Espinosa, I. Arroyo-Bergera, L. Herrera-Valdez, L. Dorronsoro-Dorronsoro.

Department of Preventive Medicine, University of Valencia, University Jaume I, Conselleria de Sanitat de la Generalitat Valenciana, Valencia, Spain: JI. González, J.V. Sorlí, O. Portolés, R. Fernández-Carrión, C. Ortega-Azorín, R. Barragán, E.M. Asensio, O. Coltell, R. Martínez-Lacruz, I. Giménez-Alba, C. Sáiz, R. Osma, E. Férriz, I. González-Monje, P. Guillém-Sáiz, F. Giménez-Fernández, L. Quiles, P. Carrasco, A. Carratalá-Calvo, C. Valero-Barceló, C. Mir, S. Sánchez-Navarro, J. Navas, I. González-Gallego, L. Bort-Llorca, L. Pérez-Ollero, M. Giner-Valero, R. Monfort-Sáez, J. Nadal-Sayol, V. Pascual-Fuster, M. Martínez-Pérez, C. Riera, M.V. Belda, A. Medina, E. Miralles, M.J. Ramírez-Esplugues, M. Rojo-Furió, G. Mattingley, M.A. Delgado, M.A. Pages, Y Riofrío, L. Abuomar, N. Blasco-Lafarga, R. Tosca, L. Lizán, A.M Valcarce, M.D. Medina, S. de Valcárcel, N. Tormo, O. Felipe-Román, S. Lafuente, E.I. Navío, G. Aldana, J.V. Crespo, J.L. Llosa, L. González-García, R. Raga-Marí.

University Rovira i Virgili, Reus, Spain: R. Pedret Llaberia, R. Gonzalez, R. Sagarra Álamo, F. París Palleja, J. Balsells, J.M. Roca, T. Basora Gallisa, J. Vizcaino, P. Llobet Alpizarte, C. Anguera Perpiñá, M. Llauradó Vernet, C. Caballero, M. Garcia Barco, M.D. Morán Martínez, J. García Rosselló, A. Del Pozo, C. Poblet Calaf, P. Arcelin Zabal, X. Floresví, M. Ciutat Benet, A. Palau Galindo, J.J. Cabré Vila, F. Dolz Andrés, J. Boj Casajuana, M. Ricard, F. Saiz, A. Isach, M. Sanchez Marin Martinez, M. Bulló, N. Becerra-Tomás, G. Mestres, J. Basora, G. Mena-Sánchez, L. Barrubés Piñol, M. Gil Segura, C. Papandreou, N. Rosique Esteban, S. Chig, I. Abellán Cano, V. Ruiz García, A. Salas-Huetos, P. Hernandez, S. Canudas, L. Camacho-Barcia, J. García-Gavilán, A. Diaz.

Cardiovascular Risk and Nutrition Research Group, Servicio de Endocrinología, IMIM (Hospital del Mar Medical Research Institute), Barcelona. Departament de Medicina, Universitat Autònoma de Barcelona, Barcelona, Spain: O. Castañer, M.A. Muñoz, M.D. Zomeño, A. Hernaéz, L. Torres, M. Quifer, R. Llimona, L.A. Gal, A. Pérez, M. Farràs, R. Elosua, J. Marrugat, J. Vila, I. Subirana, S. Pérez, M.A. Muñoz, A. Goday, J.J. Chillaron Jordan, J.A. Flores Lerroux, D. Benaiges Boix, M. Farré, E. Menoyo, D. Muñoz-Aguayo, S. Gaixas, G. Blanchart, A. Sanllorente, M. Soria, J. Valussi, A. Cuenca, L. Forcano, A. Pastor, A. Boronat, S. Tello, M. Cabañero, L. Franco, H. Schröder, R. De la Torre, C. Medrano, J. Bayó, M.T. García, V. Robledo, P. Babi, E. Canals, N. Soldevila, L. Carrés, C. Roca, M.S. Comas, G. Gasulla, X. Herraiz, A. Martínez, E. Vinyoles, J.M. Verdú, M. Masague Aguade, E. Baltasar Massip, M. Lopez Grau, M. Mengual, V. Moldon, M. Vila Vergaz, R. Cabanes Gómez, Ciurana, M. Gili Riu, A. Palomeras Vidal.

Miguel Hernández University, Alicante, Spain: Eva M. Navarrete Muñoz, Manuela García de la Hera, Sandra González Palacios, Laura Torres Collado, Laura Compañ Gabucio, Alejandro Oncina Canovas, Leyre Notario-Barandiaran, Domingo Orozco Beltran, Salvador Pertusa Martínez, Alberto Asensio, Inma Candela García, Juan Manuel Zazo, Cristina Gisbert Sellés, Noelia Fernandez Brufal, Josefa Román Maciá, Concepción Sánchez Botella, Rosario Lloret Macián, Ana Pastor Morell, Esperanza Alonso Bartolomé, Juan José Ballester Baixauli, Mª Teresa Cano Sánchez, Blanca Esther Ayús Rojo, Eugenio Pedro Cases Pérez, Cristina Tercero Maciá, Luis Alfredo Mira Castelló, Inés de los Angeles García García, Marta Jordá Ballesta, Concepción Pastor Polo, Estanislao Puig Agulló

Hospital Son Espases (HUSE) and Institute for Health Research Illes Balears (IdISBa), Palma de Mallorca, Spain: M. Fiol, M. Moñino, A. Colom, J. Konieczna, M. Morey, R. Zamanillo, A.M.

Galmés, V. Pereira, M.A. Martín, A. Yáñez, J. Llobera, J. Ripoll, R. Prieto, F. Grases, A. Costa, C. Fernández-Palomeque, E. Fortuny, M. Noris, S. Munuera, F. Tomás, F. Fiol, A. Jover, J.M. Janer, C. Vallespir, I. Mattei, N. Feuerbach, M. del Mar Sureda, S. Vega, L. Quintana, A. Fiol, M. Amador, S. González, J. Coll, A. Moyá.

Department of Nutrition, Food Sciences, and Physiology, Center for Nutrition Research, University of Navarra, Pamplona, Spain: I. Abete, I. Cantero, C. Cristobo, I. Ibero-Baraibar, M. Zulet, J. Ágreda-Martínez, M.D. Lezáun-Burgui, N. Goñi-Ruiz, R. Bartolomé-Resano, E. Cano-Cáceres, T. Elcarte-López, E. Echarte-Osacain, B. Pérez-Sanz, I. Blanco-Platero, S.A. Andueza-Azcárate, A. Gimeno-Aznar, E. Ursúa-Sesma, B. Ojeda-Bilbao, J. Martinez-Jarauta, L. Ugalde-Sarasa, B. Rípodas-Echarte, M.V. Güeto-Rubio.

Department School of Nursing, School of Health Sciences, University of Málaga-IBIMA, Málaga, Spain: F.J. Barón-López, J.C. Fernández García, N. Pérez-Farinós, N. Moreno-Morales, M. del C. Rodríguez-Martínez, J. Pérez-López, J.C. Benavente-Marín, E. Crespo Oliva, E. Contreras Fernández, F.J. Carmona González, R. Carabaño Moral, S. Torres Moreno, M.V. Martín Ruíz, M. Alcalá Cornide, V. Fuentes Gómez.

Lipids and Atherosclerosis Unit, Department of Internal Medicine, Maimonides Biomedical Research Institute of Cordoba (IMIBIC), Reina Sofia University Hospital, University of Cordoba, Cordoba, Spain: J. Criado García, A.I. Jiménez Morales, N. Delgado Casado, A. Ortiz Morales, J.D. Torres Peña, F.J. Gómez Delgado, F. Rodríguez Cantalejo, J. Caballero Villaraso, J.F. Alcalá, P.J. Peña Orihuela, G. Quintana Navarro.

Hospital Clinic, Institute for Biomedical Research August Pi i Sunyer, Barcelona, Spain: R. Casas, M. Domenech, C. Viñas, S. Castro-Barquero, A.M. Ruiz-León, M. Sadurní, G. Frontana, P. Villanueva, M. Gual, R. Soriano, M. Camafort, C. Sierra, E. Sacanella, A. Sala-Vila, J. M. Cots, I. Sarroca, M. García, N. Bermúdez, A. Pérez, I. Duaso, A. de la Arada, R. Hernández, C. Simón, M.A. de la Poza, I. Gil, M. Vila, C. Iglesias, N. Assens, M. Amatller, LL. Rams, T. Benet, G. Fernández, J. Teruel, A. Azorin, M. Cubells, D. López, J.M. Llovet, M.L. Gómez, P. Climente, L. de Paula, J. Soto, C. Carbonell, C. Llor, X. Abat, A. Cama, M. Fortuny, C. Domingo, A. I. Liberal, T. Martínez, E. Yañez, M. J. Nieto, A. Pérez, E. Lloret, C. Carrazoni, A. M. Belles, C. Olmos, M. Ramentol, M. J. Capell, R. Casas, I. Giner, A. Muñoz, R. Martín, E. Moron, A. Bonillo, G. Sánchez, C. Calbó, J. Pous, M. Massip, Y. García, M.C. Massagué, R. Ibañez, J. Llaona, T. Vidal, N. Vizcay, E. Segura, C. Galindo, M. Moreno, M. Caubet, J. Altirriba, G. Fluxà, P. Toribio, E. Torrent, J. J. Anton, A. Viaplana, G. Vieytes, N. Duch, A. Pereira, M. A. Moreno, A. Pérez, E. Sant, J. Gené, H. Calvillo, F. Pont, M. Puig, M. Casasayas, A. Garrich, E. Senar, A. Martínez, I. Boix, E. Sequeira, V. Aragunde, S. Riera, M. Salgado, M. Fuentes, E. Martín, A. Ubieto, F. Pallarés, C. Sala, A. Abilla, S. Moreno, E. Mayor, T. Colom, A. Gaspar, A. Gómez, L. Palacios, R. Garrigosa.

Departament of Preventive Medicine and Public Health, University of Granada, Granada, Spain: L. García Molina, B. Riquelme Gallego, N. Cano Ibañez, A. Maldonado Calvo, A. López Maldonado, E.M. Garrido, A. Baena Dominguez, F. García Jiménez, E. Thomas Carazo, A. Jesús Turnes González, F. González Jiménez, F. Padilla Ruiz, J. Machado Santiago, M.D. Martínez Bellón, A. Pueyos Sánchez, L. Arribas Mir, R. Rodríguez Tapioles, F. Dorador Atienza, L. Baena Camus, C. Osorio Martos, D. Rueda Lozano, M. López Alcázar, F. Ramos Díaz, M. Cruz Rosales Sierra, P. Alguacil Cubero, A. López Rodriguez, F. Guerrero García, J. Tormo Molina, F. Ruiz Rodríguez.

OSI ARABA, University Hospital Araba, Vitoria, Spain: J. Rekondo, I. Salaverria, A. Alonso-Gómez, M.C. Belló, A. Loma-Osorio, L. Tojal, P. Bruyel, L. Goicolea, C. Sorto, A. Casi Casanellas,

M.L. Arnal Otero, J. Ortueta Martínez De Arbulo, J. Vinagre Morgado, J. Romeo Ollora, J. Urraca, M.I. Sarriegui Carrera, F.J. Toribio, E. Magán, A. Rodríguez, S. Castro Madrid, M.T. Gómez Merino, M. Rodríguez Jiménez, M. Gutiérrez Jodra, B. López Alonso, J. Iturralde Iriso, C. Pascual Romero, A. Izquierdo De La Guerra.

Research Group on Community Nutrition & Oxidative Stress, University of Balearic Islands, Palma de Mallorca, Spain: M. Abbate, I. Aguilar, E. Angullo, A. Arenas, E. Argelich, M.M. Bibiloni, Y. Bisbal, C. Bouzas, C. Busquets, X. Capó, S. Carreres, A. De la Peña, L. Gallardo, J.M. Gámez, B. García, C. García, A. Julibert, I. Llompart, C.M. Mascaró, D. Mateos, S. Montemayor, A. Pons, T. Ripoll, T. Rodríguez, E. Salaberry, A. Sureda, S. Tejada, L. Ugarriza, L. Valiño.

Virgen de la Victoria Hospital, University of Málaga, Málaga, Spain: M.R. Bernal López, M. Macías González, J. Ruiz Nava, J.C. Fernández García, A. Muñoz Garach, A. Vilches Pérez, A. González Banderas, J. Alcaide Torres, A. Vargas Candela, M. León Fernández, R. Hernández Robles, S. Santamaría Fernández, J.M. Marín, S. Valdés Hernández, J.C. Villalobos, A. Ortiz.

University of Las Palmas de Gran Canaria, Las Palmas, Spain: J. Álvarez-Pérez, E.M. Díaz Benítez, F. Díaz-Collado, A. Sánchez-Villegas, J. Pérez-Cabrera, L.T. Casañas-Quintana, R.B. García-Guerra, I. Bautista-Castaño, C. Ruano-Rodríguez, F. Sarmiento de la Fe, J.A. García-Pastor, B. Macías-Gutiérrez, I. Falcón-Sanabria, C. Simón-García, A.J. Santana-Santana, J.B. Álvarez-Álvarez, B.V. Díaz-González, J.M. Castillo Anzalas, R.E. Sosa-Also, J. Medina-Ponce.

Biomedicine Institute (IBIOMED); University of León, and Primary Health Care Management of León (Sacyl), León, Spain: S. Abajo Olea, A. Adlbi Sibai, A. Aguado Arconada, L. Álvarez, E. Carriedo Ule, M. Escobar Fernández, J.I. Ferradal García, J.P. Fernández Vázquez, M. García González, C. González Donquiles, C. González Quintana, F. González Rivero, M. Lavinia Popescu, J.I. López Gil, J. López de la Iglesia, A. Marcos Delgado, C. Merino Acevedo, S. Reguero Celada, M. Rodríguez Bul, L. Vilorio-Marqués.

Department of Family Medicine, Primary Care Division of Sevilla, Sevilla, Spain: J.M. Santos-Lozano, L. Miró-Moriano, C. Domínguez-Espinaco, S. Vaquero-Díaz, F.J. García-Corte, A. Santos-Calonge, C. Toro-Cortés, N. Pelegrina-López, V. Urbano-Fernández, M. Ortega-Calvo, J. Lozano-Rodríguez, I. Rivera-Benítez, M. Caballero-Valderrama, P. Iglesias-Bonilla, P. Román-Torres, Y. Corchado-Albalat, E. Mayoral-Sánchez.

Department of Endocrinology, Foundation Jiménez-Díaz, Madrid, Spain: A.I. de Cos, S. Gutierrez, S. Artola, A. Galdon, I. Gonzalo.

Lipids and Vascular Risk Unit, Internal Medicine, University Hospital of Bellvitge, Hospitalet de Llobregat, Barcelona, Spain: A. Galera, M. Gimenez-Gracia, R. Figueras, M. Poch, R. Freixedas, F. Trias, I. Sarasa, M. Fanlo, H. Lafuente, M. Liceran, A. Rodriguez-Sanchez, C. Pallarols, J. Monedero, X. Corbella, E. Corbella.

Department of Endocrinology, IDIBAPS, Hospital Clinic, University of Barcelona, Barcelona, Spain: A. Altés, I. Vinagre, C. Mestres, J. Viaplana, M. Serra, J. Vera, T. Freitas, E. Ortega, I. Pla.

Nutritional Genomics and Epigenomics Group, Institute IMDEA-Food, CEI UAM+CSIC, Madrid, Spain: L. Berninches, M.J. Concejo, J. Muñoz, M. Adrián, Y. de la Fuente, C. Albertos, E. Villahoz, M.L. Cornejo., A. Montero., J. Tapia. , C. Cuesta

Division of Preventive Medicine, University of Jaén, Jaén, Spain: J.J. Gaforio, S. Moraleda, N. Liétor, J.I. Peis, T. Ureña, M. Rueda, M.I. Ballesta.

Department of Endocrinology and Nutrition, Institute for Health Research Hospital Clínico San Carlos (IdISSC), Madrid, Spain: C. Moreno Lopera, C. Aragoneses Isabel, M.A. Sirur Flores, M. Ceballos de Diego, T. Bescos Cáceres, Y. Peña Cereceda, M. Martínez Abad, R. Cabrera Vela, M. González Cerrajero, M.A. Rubio Herrera, M. Torrego Ellacuría, A. Barabash Bustelo, M. Ortiz Ramos, U. Garin Barrutia.

Department of Basic and Clinical Psychology and Psychobiology, University Jaume I, Castellón de la Plana, Spain: R. Baños, A. García-Palacios.

Department of Biochemistry and Molecular Biology, Faculty of Medicine and Odontology, Service of Clinical Analysis, University Hospital Dr. Peset. University of Valencia, Valencia, Spain: C. Cerdá Micó, N. Estañ Capell, A. Iradi, M. Fandos Sánchez.

Department of Preventive Medicine, University of Malaga, Malaga, Spain: J. Fernández-Crehuet Navajas, M. Gutiérrez Bedmar, A. García Rodriguez, A. Mariscal Larrubia, M. Carnero Varo, C. Muñoz Bravo.
